# “You Are What You Eat”: How Fungal Adaptation Can Be Leveraged toward Myco‐Material Properties

**DOI:** 10.1002/gch2.202300140

**Published:** 2023-11-08

**Authors:** Alicia Vivas Hernando, Wenjing Sun, Tiffany Abitbol

**Affiliations:** ^1^ Institute of Materials (IMX) École Polytechnique Fédérale de Lausanne (EPFL) Lausanne 1015 Switzerland

**Keywords:** biocomposites, environment, fungi, genetics, mycelium, myco‐materials

## Abstract

Fungi adapt to their surroundings, modifying their behaviors and composition under different conditions like nutrient availability and environmental stress. This perspective examines how a basic understanding of fungal genetics and the different ways that fungi can be influenced by their surroundings can be leveraged toward the production of functional mycelium materials. Simply put, within the constraints of a given genetic script, both the quality and quantity of fungal mycelium are shaped by what they eat and where they grow. These two levers, encompassing their global growth environment, can be turned toward different materials outcomes. The final properties of myco‐materials are thus intimately shaped by the conditions of their growth, enabling the design of new biobased and biodegradable material constructions for applications that have traditionally relied on petroleum‐based chemicals.This perspective highlights aspects of fungal genetics and environmental adaptation that have potential materials science implications, along the way touching on key studies, both to situate the state of the art within the field and to punctuate the viewpoints of the authors. Finally, this work ends with future perspectives, reinforcing key topics deemed important to consider in emerging myco‐materials research.

## Graphical abstract

This perspective aims to critically explore the different levers that can be turned to obtain functional and reliable myco‐materials engineered from fungi. These levers include fungal species, encompassing their specific set of genetic information, as well as global environmental cues, encompassing aspects of nutrition, that can be used to coax end material properties toward a desired outcome via fungal adaptation.

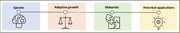

## 1 Introduction

In the face of increasingly disturbing environmental calamities, the topic of sustainability has climbed to the top of nearly everyone's radar.^[^ ^1^, ^2^ ^]^ At its heart, sustainability defends a system where current needs are met without compromising those of future generations.^[^ ^3^ ^]^ Considering humankind's disproportionate use of resources^[^ ^2^, ^4^ ^]^ and excessive generation of waste,^[^ ^2^ ^]^ it is undeniable that meeting sustainability targets, such as those outlined in the UN Sustainable Development Goals (UN SDGs), calls for a radical rethinking of the materials life cycle.

Materials are at the forefront of the sustainability revolution (for example, UN SDG 12 on responsible consumption and production), playing a crucial role in providing environmentally‐sound solutions that can help tackle today's climate and energy crises.^[^ ^5^, ^6^, ^7^, ^8^ ^]^ Until recently, the perception of inexhaustible resources has prevailed, fueling unchecked exploitation to meet growing demands.^[^ ^9^ ^]^ Reflective of humanity's misuse of resources, since the 1970s we have been living in an ecological overshoot, meaning that the demands placed on the planet's ecosystem and resources surpass its regenerative capacity.^[^ ^4^ ^]^ Thus, abandoning the “take‐make‐discard” status‐quo relationship with materials is essential for a sustainable future.^[^ ^8^ ^]^

In this context, fungal biotechnology plays a role in the transition to a circular economy, with its potential breadth aligning with many of the 17 UN SDGs.^[^ ^10^ ^]^ Fungal fermentation as a cell factory for food production and secondary metabolites for the chemical, pharmaceutical, and pulp and paper industries is well known, however its application as a versatile biomaterial platform has only emerged over the past 15 years or so. Myco‐materials are derived from mycelium, the vegetative body of fungi, composed of a mass of filamentous tubular constructions called hyphae.^[^ ^11^ ^]^ Myco‐materials can consist of mycelium either as their main or sole constituent, as in the case of pure mycelium materials, or in lesser amounts within mycelium‐bound composites. These materials are produced via the metabolism of filamentous fungi, many of which have evolved to decompose recalcitrant biomass, preventing soil, forests, and waterways from being overrun with plant litter and animal tissue.^[^ ^12^ ^]^ As such, fungal mycelium growth is inherently suited for the repurposing of lignocellulosic industrial by‐products, yielding mycelium‐lignocellulose composites by building up cohesive 3D network structures.^[^ ^13^ ^]^ The transformative and disruptive qualities of these materials has led to the commercial development of animal‐free leathers,^[^ ^14^ ^]^ lightweight foams for acoustic insulation and packaging,^[^ ^15^ ^]^ with companies as recognized as Dell^[^ ^16^ ^]^ and Ikea^[^ ^17^ ^]^ employing myco‐composite foams as substitutes for traditional polystyrene‐derived product cushioning.

To fully exploit the capabilities of these organisms from a materials science perspective, we think it's important to consider how the interplay between mycelium genetics and growth conditions translates into material properties. This thinking is reminiscent of the concept of  “nature versus nurture”, a term used by psychologists and biologists as a way to address whether behavioral traits are inherited (i.e., nature) or acquired (i.e., nurture).^[^ ^18^ ^]^ The old adage “you are what you eat” also holds up in the context of mycelium – through complex networks of enzymes and cofactors, organisms evolve and adapt their metabolism according to available nutrient sources and external conditions.^[^ ^19^, ^20^ ^]^ Fungal genetics and their growth environment, including the characteristics of the substrate, come together to define the composition, quantity, and quality of the resulting biomass and, consequently, the properties of the final myco‐material.^[^ ^21^ ^]^

The aim of this perspective is to dive deep into how environment (“nature versus nurture”) and diet (“you are what you eat”), both of which can be lumped into the broad category of global environment, can be leveraged in cultivating fungi as a source of materials, to better enable the production of myco‐materials with reliable, robust, and useful properties. First, we will describe what happens when mycelium grows, highlighting aspects related to cell wall architecture and fungal metabolism that are important from a materials standpoint. Then, we will draw the reader's attention to specific examples from the literature where fungal genetics and growth conditions are found to impact aspects relevant to materials science, whether or not materials applications were originally intended. The balance of nutritive and non‐nutritive additives (you are what you eat!) relating to the composition and structure of fermented products, is especially considered. Finally, we end with our perspectives on the current state of the field and where we see opportunities in developing functional materials from fungal mycelium.

## 2 Aspects of Fungal Cell Wall Composition, Metabolism, and Adaptation

**Figure 1:**
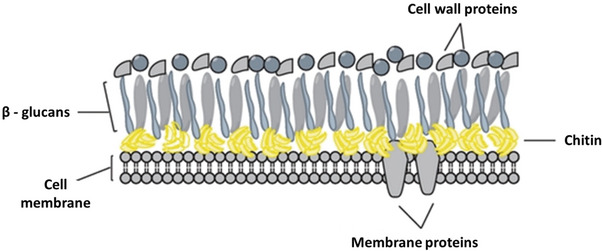
Schematic depiction of the fungal cell wall. The inner layer is formed by crosslinked chitin and β‐glucans, while the outer layer is mannoprotein‐ and glycoprotein‐rich. Adapted from: Vega et al., 2012.^[^ ^22^ ^]^

As mentioned above, myco‐materials are composed of mycelium to varying amounts, depending on the approach, and are made up of hyphae. The tubular cell wall of hyphae is formed by elongated cells, separated by porous cross walls, or septa, with growth occurring through the extension of the cell membrane from the cell tips.^[^ ^11^ ^]^ Fungi rely on their cell wall to maintain structural integrity and cellular viability, and to mediate their interactions with the external environment. Common to most fungal types, the cell wall consists of layers of chitin/chitosan, β(1‐3) and other alkali‐insoluble glucans, various cell wall proteins (**Figure** **1**), and smaller amounts of lipids and pigments.^[^ ^11^ ^]^ Chitin, a load‐bearing polysaccharide, is composed of β(1‐4)‐linked N‐acetyl‐2‐amino‐2‐deoxy‐D‐glucose units. Contiguous chitin chains in the cell wall impart tensile strength to the hyphae via their assembly into hydrogen‐bonded antiparallel microfibrils.^[^ ^23^ ^]^ Still, glucans are the most prevalent polymers in the cell wall, where their monomers connect through α or β linkages and impart mechanical integrity to the hyphae.^[^ ^23^ ^]^ The external part of the cell wall varies among class and genus but usually contains alkali‐soluble glucans and proteins, mainly in the form of glycoproteins with N‐ and O‐linked carbohydrates, which impart ductility to the mycelium body.^[^ ^24^ ^]^ One of the main functions of the proteins is the extracellular digestion of lignocellulosic food sources, which is accomplished through the secretion of specific enzymes such as laccases, peroxidases, oxidases, cellulases, and different glycosidases.^[^ ^25^ ^]^ Another important group of proteins in the fungal cell wall are hydrophobins, which promote aerial development and attachment to solid supports and confer hydrophobicity via their assembly into amphipathic membranes.^[^ ^26^, ^27^ ^]^

Fungi require organic sources of carbon and energy.^[^ ^28^ ^]^ Being heterotrophic, they extract the energy stored in the bonds of organic compounds within living or dead organisms, using primary metabolic pathways similar to those in animals (i.e., glycolytic, tricarboxylic acid, and pentose phosphate pathways).^[^ ^28^, ^29^ ^]^ Sugars are the preferred form of carbon and energy, requiring the least energy to assimilate and being readily digested by fungi through both active and passive systems.^[^ ^28^ ^]^ However, since easily consumable monosaccharides such as glucose rarely exist in nature, fungi depend mostly on the degradation of complex insoluble carbohydrates, like those from cellulose or animal tissues, to get the nutrients they need to grow.^[^ ^28^, ^29^ ^]^ For this purpose, they are able to secrete enzymes that can degrade a wide variety of polymers, possessing global transcription factors that can regulate the expression of various pathways in response to fluctuating nutrient levels.^[^ ^28^, ^29^ ^]^

Lignocellulosic biomass, composed of mostly cellulose, hemicellulose, and lignin, stands out as the primary carbon and energy supply in natural environments. Fungi employ a combination of oxidative and hydrolytic enzymes to extracellularly degrade lignocellulosic sources: oxidative enzymes, such as lignin peroxidases, manganese peroxidases, and laccases, degrade lignin and open phenyl rings, whereas hydrolytic enzymes, such as endoglucanases, β‐glucosidases, and cellobiohydrolases degrade polysaccharides.^[^ ^28^, ^30^ ^]^ Another important nutrient needed for the survival of fungi is nitrogen. Unlike animals that obtain amino acids from their food, fungi can synthesize the amino acids necessary for their development from inorganic nitrogen sources available in the surrounding substrates, such as from soil nitrates, ammonia, or even fixed from the air for some species.^[^ ^28^, ^31^ ^]^ Other essential nutrients include macronutrients such as phosphorus, potassium, and sulfur, and micronutrients such as manganese, iron, zinc, and copper^[^ ^28^ ^]^ Fungi also synthesize numerous chemical compounds via their secondary metabolism, and while the biological roles of these secondary metabolites remains elusive in some instances, these often potent molecules have been commercially exploited in various industries.^[^ ^40^, ^41^ ^]^

Fungi also require a specific set of environmental conditions to survive. Most fungi are aerobic organisms, meaning that they need oxygen as it's essential for the generation of energy.^[^ ^32^ ^]^ Different species display significant variability in their response to changes in oxygen pressure, which probably relates to differences in their degradation pathways.^[^ ^33^ ^]^ At various stages of growth, the requirements for air composition differ; for instance, aerobic fungi typically require lower CO_2_ concentrations for fruiting than during their mycelial expansion phase.^[^ ^34^ ^]^ Water is another essential chemical required for fungi to grow, and a high water activity is generally required for the growth of most fungal species, with few exceptions.^[^ ^29^ ^]^ The internal hydrostatic pressure (turgor) of fungal hyphae provides both mechanical support and the driving impetus for expansion and substrate penetration, and it varies with changes in the growth environment.^[^ ^35^ ^]^ Fungi maintain high turgor pressure when exposed to hypertonic osmotic conditions, or when grown on rigid substrates or in nutrient‐rich environments. In contrast, they reduce their turgor pressure when exposed to low osmotic conditions or when grown in loose substrates, such as soil or nutrient‐poor environments.^[^ ^36^, ^37^, ^38^ ^]^ The temperature suitable for fungal growth is generally ≈25 °C,^[^ ^29^ ^]^ although optimal temperatures can differ across species, nutritional conditions, and other growth‐related parameters.^[^ ^39^, ^40^, ^41^ ^]^ A small group of fungi are able to extend their growth to temperatures as high as 62 °C (thermophilic fungi)^[^ ^42^ ^]^ or as low as 0 °C (cryophilic fungi),^[^ ^42^ ^]^ but extreme temperatures can cause cell damage through the denaturation of proteins and nucleic acids.^[^ ^43^ ^]^ Most fungi can grow within a wide pH range (pH 3–8), with some able to thrive under more extreme pH conditions.^[^ ^29^ ^]^ They have the ability to sense the pH of their surroundings and adjust gene expression to regulate and sustain a stable internal pH.^[^ ^44^, ^45^, ^46^ ^]^ Although light is not used by fungi to obtain energy, it serves as a critical source of information and impacts their growth and behavior, including metabolism and reproduction.^[^ ^47^, ^48^ ^]^ For example, in certain fungal species, the presence of light is necessary to trigger spore production and/or the development of fruiting bodies, which is one of the reasons that myco‐materials are usually grown in the dark.^[^ ^47^ ^]^ Extreme radiation such as UV‐B light, gamma rays, and microwaves are toxic to many fungal species, but more pigmented fungi tend to be more resilient to these conditions.^[^ ^49^ ^]^

An interesting feature of fungi is their adaptability in modifying their growth patterns in response to changes in substrate and environment. This adaptability includes morphological changes,^[^ ^50^, ^51^, ^52^, ^53^, ^54^, ^55^, ^56^, ^57^ ^]^ adjusting their growth direction,^[^ ^47^, ^58^, ^59^, ^60^, ^61^ ^]^ alternating between different reproductive strategies,^[^ ^62^, ^63^, ^64^, ^65^ ^]^ or altering their metabolic pathways in response to environmental cues.^[^ ^48^, ^66^ ^]^ The impressive adaptability and resilience of fungi have been extensively studied and applied in numerous industrial contexts. Their ability to survive in a wide range of habitats, including soil, freshwater, and marine environments, and to secrete enzymes that can break down hazardous substances, makes them interesting from a bioremediation perspective. Fungi can convert toxic and recalcitrant pollutants into environmentally benign products in a process that is called myco‐remediation.^[^ ^67^ ^]^ In fact, among all microbes, fungi are often the most efficient removers of heavy metals due to their high tolerance to heavy metals, large surface area to volume ratio, and greater robustness compared to bacteria and algae. Fungi employ both passive and active mechanisms for the passivation and removal of environmental toxins, specifically biosorption, a passive, metabolically‐independent process achievable by both live cells and dead biomass; bioaccumulation, an active, metabolically‐dependent process occurring in live cells; and biovolatilization, the active conversion of inorganic and organic compounds into volatile derivatives through intracellular enzymatic reactions.^[^ ^68^ ^]^ These characteristics can also be extended to organic compounds, enabling fungi to degrade pollutant chemicals, including polycyclic aromatic hydrocarbons (PAHs), agricultural wastes (pesticides and herbicides), phthalates, dyes or detergents, and pharmaceutical waste.^[^ ^68^ ^]^ For example, some filamentous fungi have demonstrated the ability to remove PAHs and pesticides from contaminated soil.^[^ ^78^ ^]^ Similarly, fungi serve as biological control agents in agriculture, producing compounds that repel pests and disease.^[^ ^69^ ^]^ The biotech industry has utilized the adaptability of fungi to produce a variety of enzymes, proteins, and other biologically active compounds with diverse applications in fields like food,^[^ ^70^ ^]^ biofuels,^[^ ^71^ ^]^ and medicine.^[^ ^72^ ^]^

## 3 Modifications for Myco‐Materials

**Figure 2:**
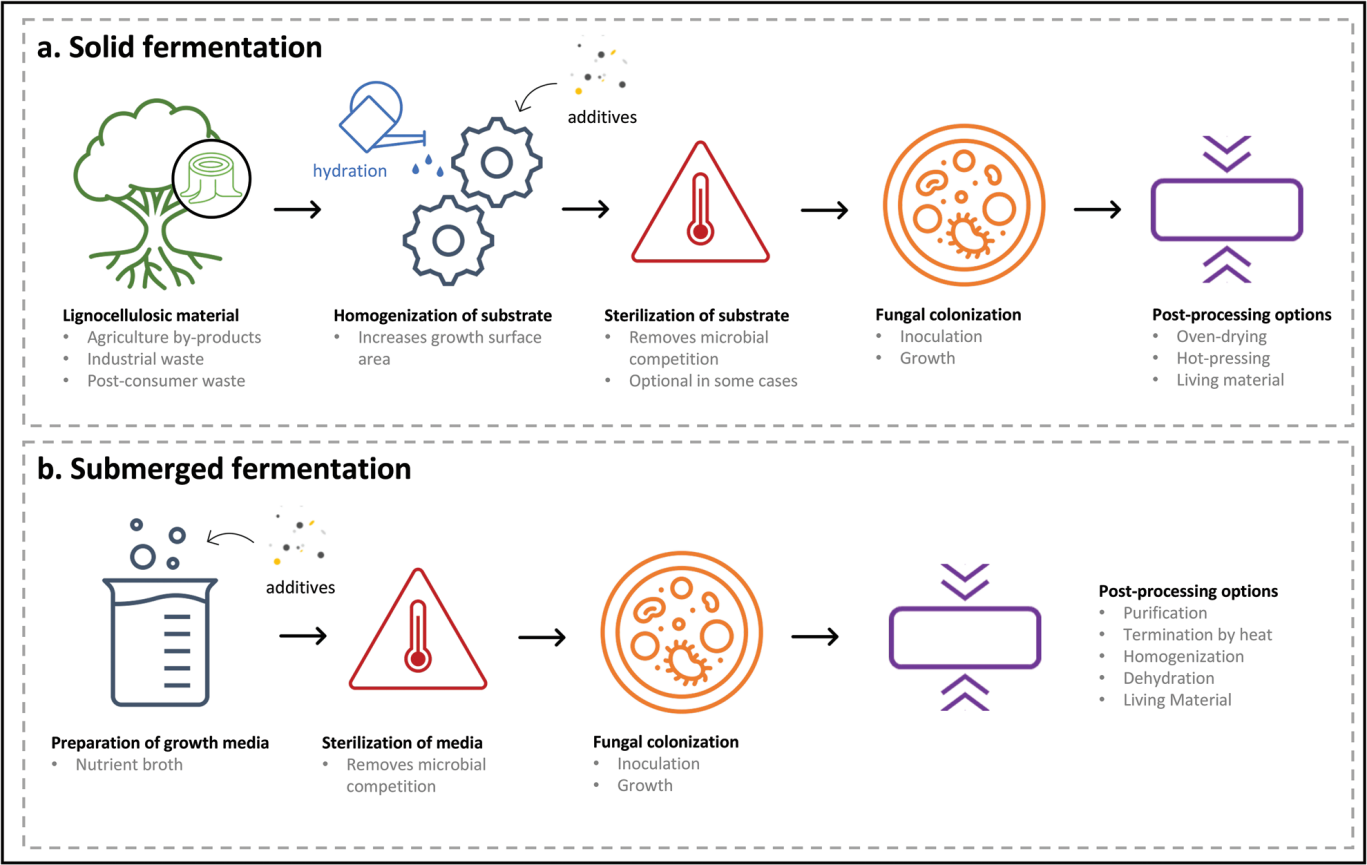
General processing scheme of myco‐materials made through a) solid and b) submerged fermentation.

Myco‐materials are the result of mycelium colonization on defined growth substrates, whether soluble, insoluble, or a mix. The most common approach to date is solid fermentation, where solid substrates, such as lignocellulosic chips, fibers, and/or particulates, serve as a food source for the mycelium, being either partially or completely consumed. Most commonly, this results in myco‐composites when the solid substrate becomes integrated and bound within the mycelium network,^[^ ^15^ ^]^ or in pure mycelium materials when the mycelium is grown on top of the solid substrate and separated from it after growth.^[^ ^73^ ^]^ Adding an element of sustainability, these substrates are typically sourced from agricultural by‐products, industrial waste, or post‐consumer waste.^[^ ^74^ ^]^ In addition to solid fermentation, mycelium‐based materials have been produced by submerged fermentation, where the nutrients and any other additives to the growth are provided within a liquid medium. In this method, the mycelium often forms discrete pellets that can be processed in different ways, for instance, by blending into a slurry and draining into films,^[^ ^75^ ^]^ or by extracting compounds from fungal biomass, as is the case with fungal nanochitin.^[^ ^76^ ^]^ **Figure** **2** illustrates the general process for myco‐material production by solid and submerged fermentation, however we wish to emphasize that these steps are intended as rough guidelines, especially concerning termination, post‐processing, and materials formation.

Many properties of myco‐materials depend on the quantity and quality of the different components within the cell wall of the fungi, aspects largely influenced by fungal species, substrate, growth conditions, and growth approach. Within fields such as biotechnology and mycology, the influence of fungal genetics and growth conditions has been studied extensively,^[^ ^28^, ^77^ ^]^ however, our review of the literature has revealed few direct connections in the context of engineered materials. Altering the biological characteristics of fungi, either through genetic engineering or by modifying growth conditions, can serve as powerful tools to engineer fungal material properties and potentially enhance the efficiency of fungal biomaterial production.

## 4 Fungal Species

In pure myco‐materials, mechanical properties seem most affected by the fungal species, linked to the morphology and properties of the hyphae. In contrast, in myco‐composites, the roles of mycelium and substrate vary with the composite systems (discussed in more detail below).^[^ ^15^, ^78^ ^]^ However, within a specific species, growth conditions (to be discussed later) can significantly influence hyphal characteristics. The most widely investigated species for myco‐composites are mostly basidiomycetes, such as *Pleurotus ostreatus* (*P. ostreatus*), *Ganoderma lucidum* (*G. lucidum*) and *Trametes versicolor* (*T. versicolor*),^[^ ^74^ ^]^ while a broader array of species has been explored in pure mycelium materials from submerged fermentation.^[^ ^75^, ^79^, ^80^ ^]^

**Figure 3:**
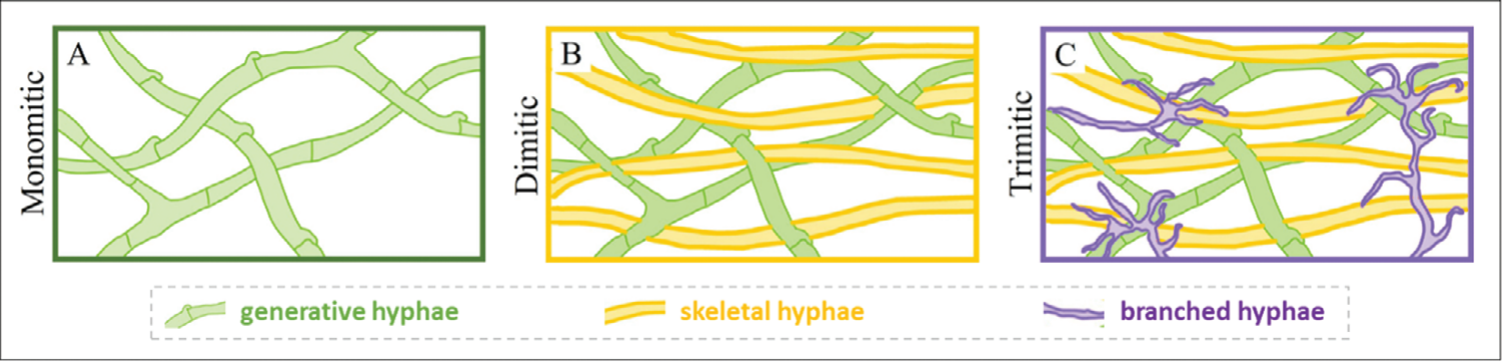
Schematic of suggested hyphal systems. Adapted from: Porter and Naleway, 2022.^[^ ^81^ ^]^

Mycelium hyphae from basidiomycetes have been categorized into three types based on their morphology: generative, skeletal, and binding.^[^ ^82^ ^]^ Generative hyphae are described as having thin walls and frequent septa and clamp connections, skeletal hyphae as being thick, long, and rarely branched, and binding hyphae as being thick‐walled, and branched.^[^ ^82^, ^83^ ^]^ Depending on the predominant hyphal type that is identified, the mycelium network has been classified as monomitic, dimitic, or trimitic if it comprises one (generative), two (typically generative and skeletal), or all three hyphal types, respectively,^[^ ^82^, ^84^ ^]^ as shown in **Figure** **3**.^[^ ^81^ ^]^ Several research papers attribute the differences in the material properties of myco‐composites^[^ ^85^ ^]^ and fruiting bodies^[^ ^81^ ^]^ to hyphal type, and at least one recent review, to our knowledge, has solidified this interpretation.^[^ ^86^ ^]^ However, it may be a stretch to draw too many firm conclusions from studies that don't always consider how hyphal properties can be influenced by a combination of factors, sometimes confounding, not only by species. Indeed, comparative studies between species are challenging, and inferring direct relationships, such as between hyphal type and mechanical properties, may be simplistic in the context of living organisms. Many studies maintain consistent growth conditions when comparing across species (including those cited above) and might overlook the possibility that conditions yielding a certain property outcome in one species may not yield the same in another. We also note that both Haneef et al. (pure mycelium materials by solid fermentation)^[^ ^87^ ^]^ and Attias and Abitbol et al. (mycelium‐cellulose biocomposites by submerged fermentation)^[^ ^88^ ^]^ observed changes in hyphal morphology/cell wall properties when comparing the same species grown under different conditions of nutrition, suggesting a more complex world, as might be expected for living organisms. Finally, from our perspective, the list of three hyphal types depicted in Figure 3 is not definitive, and the connection between material properties and perceived hyphal type(s), while intriguing, remains to be fully elucidated.

The degradation mechanisms inherent to the fungal species also influence myco‐composite characteristics. To obtain a matrix with good mechanical performance and stability, it is important that the cellulose contained within a lignocellulosic substrate remains largely intact. Although white‐rot fungi decompose all wood constituents, they cause little damage to the mechanical integrity of the substrate at early stages of decay.^[^ ^89^, ^90^ ^]^ Furthermore, they have the ability to develop continuous mycelium networks, which is why they generally seem to be a good fit for the production of mycelium‐bound biocomposites, compared to brown or soft‐rot species. Additionally, common substrates for myco‐composites are derived from industrial waste streams, which are typically heterogeneous and contain organic, inorganic, and synthetic components in varying proportions. Therefore, fungal species capable of colonizing and degrading a wide range of substances may provide added value to the resulting myco‐composite. For example, Wu et al. investigated the degradation performance of the white‐rot *Phanerochaete chrysosporium* (*P. chrysosporium*) against polylactic acid (PLA) and polystyrene (PS), achieving mass losses of ≈34 and 23%, respectively, after a 35‐day incubation.^[^ ^91^ ^]^ This implies that certain fungal species have the capacity to degrade plastics to some degree, a characteristic that can be exploited when using industrial or mixed residues as substrates for myco‐composites. Beyond plastic biodegradation, which is a worthy avenue in and of itself, it will be interesting to understand how a diet that includes plastic influences the properties of the grown mycelium, and whether any diet‐induced changes can be leveraged toward new properties in mycelium‐bound plastics composites.

## 5 Genetics and Cell Wall Composition

In biotech, genetic modification of fungi, especially for producing enzymes, exopolysaccharides, acids, and secondary metabolites, has been extensively researched and implemented.^[^ ^77^, ^92^ ^]^ The protein secretion pathway of fungi is divided into three main steps. First, polypeptide transfer occurs between the ribosome and the endolasmic reticulum (ER), mediated by the signal peptide recognition particle (SRP). Then, protein folding and modification occurs in the ER, aided by molecular chaperones and folding enzymes. The unfolded protein response (UPR) detects unfolded proteins, inducing chaperone and folding enzyme biosynthesis, while the ER‐associated protein degradation (ERAD) degrades misfolded proteins. Finally, correctly folded proteins are transported to the Golgi apparatus and secreted into the extracellular environment.^[^ ^92^ ^]^ Genetic engineering in filamentous fungi can be accomplished in a few different ways. Traditional genetic transformation techniques, such as protoplast‐mediated transformation (PMT), *Agrobacterium*‐mediated transformation (AMT), electroporation (EP), biolistic transformation (BT) or shock waves‐mediated transformation (SWMT), are DNA‐based approaches that target the expression or deletion of single genes or pathways.^[^ ^77^, ^92^ ^]^ Recent developments in the CRISPR‐Cas9 technology have enabled expression, suppression, and point mutations within the whole genome, expanding the scope of genetic engineering possibilities.^[^ ^92^, ^93^ ^]^ RNA‐based methods have also been studied to silence gene expression post‐transcriptionally.^[^ ^77^ ^]^

Due to their metabolic diversity, fungi are particularly well‐suited to genetic modification, allowing control over the overexpression, suppression, and introduction of specific genes, to enhance the production of desired compounds, as well as growth and degradation patterns.^[^ ^92^ ^]^ For instance, Wang et al. replaced the original signal peptide with a more efficient one, obtaining a 3.4‐fold increase in the secretion of starch‐degrading enzymes compared to the parent strains.^[^ ^94^ ^]^ Regulation of UPR and ERAD can also promote protein secretion, as in Wu et al.’s work, where glucose oxidase secretion was improved up to 1.8‐fold, which may influence fungal metabolism and promote antibacterial activity.^[^ ^95^, ^96^ ^]^ Cellulase activity was influenced similarly, as demonstrated by Han et al., where carbon catabolite repression was used to enhance cellulase production in the presence of glucose,^[^ ^97^ ^]^ or Fitz et al.’s work, where hyperbranched phenotypes and a 3‐fold improvement in cellulase secretion were reported under rac1 gene deletion.^[^ ^98^ ^]^ The above examples are just a few of the many that demonstrate how the traditional genetic engineering of fungi can be employed to guide fungal metabolism for myco‐material production, for instance in the enrichment of key enzymes that digest substrates, perhaps enabling faster growth times, higher yields, or some other materials‐relevant benefit.

Unlike myco‐composites, the properties of pure mycelium materials predominantly depend on the composition and morphology of the hyphal cell wall, for example, higher chitin contents are shown to impart stiffness and strength to resulting myco‐materials.^[^ ^87^, ^88^ ^]^ From a genetic engineering point of view, control over the composition of the fungal cell wall can be achieved by overexpressing or suppressing genes involved in specific biosynthetic pathways. For instance, genetically engineered chitinases, like those used with *Trichoderma harziamun* (*T. harziamun*) and *Beauveira bassiana* (*B. bassiana*),^[^ ^99^ ^]^ can degrade chitin and remodel the cell wall during hyphal growth,^[^ ^100^ ^]^ potentially impacting mechanical properties. Fungal chitin is naturally bound to glucan, serving as a flexible matrix and consequently providing added mechanical performance to end materials.^[^ ^101^ ^]^ Papers produced from fungal chitin extracts retaining 50–65% glucans demonstrated high tensile strength (>200 MPa) and modulus (≈7 GPa).^[^ ^102^ ^]^ Furthermore, lipids and proteins in the cell wall have been shown to act as plasticizers, imparting ductility.^[^ ^87^ ^]^ Similarly, hydrophobin gene deletion in *Schizophyllum commune* (*S. commune*) resulted in a 3 to 4‐fold improvement in Young's modulus and maximum tensile strength values.^[^ ^103^ ^]^

Because of their amphipathic properties, hydrophobins are additionally interesting as biosurfactants, possessing distinct mass transfer properties that could be useful in fields such as packaging or biomedicine,^[^ ^104^ ^]^ for instance, by providing barrier properties. Wang et al. specifically studied *S. commune* hydrophobins, demonstrating oil‐emulsifying properties and impermeability to solutes > 200 Da.^[^ ^105^ ^]^ Furthermore, as they are sulfur‐rich, hydrophobins can also potentially provide thermal stability and flame retardant properties to myco‐composites by reducing the release of volatile substances, such as levoglucosan and furan derivatives, and favoring carbonization. As an example, improved flame retardancy properties and thermally stable carbonaceous structures were obtained through the hydrophobin surface treatment of cotton fabrics, as the protein coating favored cellulose dehydration over its depolymerization.^[^ ^106^ ^]^ Thus, the possibility to engineer strains that secrete more hydrophobins may be highly interesting, however we note that the isolation of purified hydrophobins generally involves many steps and harsh solvents, all for a rather low yield,^[^ ^107^ ^]^ and is therefore perhaps unsuited for large‐scale commercial applications.

Stress on the cell wall significantly influences the morphological and compositional alterations in the fungal cell wall, activating compensatory mechanisms to maintain its integrity.^[^ ^108^, ^109^ ^]^ This regulation of compositional biosynthesis is accomplished through a complex system of multilevel signaling pathways that involve changes in both gene expression and protein activity.^[^ ^24^, ^110^, ^111^ ^]^ Conditions of stress can be leveraged to increase cell wall rigidity, and therefore strengthen or otherwise modify the properties of mycelium materials, or to increase the yield of chitinous extracts. Fungi commonly employ chitin hyperaccumulation as a reinforcement strategy when their cell walls are weakened by mutation,^[^ ^112^, ^113^ ^]^ or otherwise modified.^[^ ^114^, ^115^ ^]^ For instance, exposing *Saccharomyces cerevisae* (*S. cerevisae*) to the α‐factor mating pheromone resulted in an 11‐fold increase in plasma membrane chitin levels.^[^ ^116^ ^]^ Similarly, increased chitin synthesis in *Aspergillus niger* (*A. niger*) cell walls was observed when subjected to both cell wall‐disturbing compounds and β‐glucan synthase inhibitors.^[^ ^108^ ^]^ Stress may also induce changes in the overall conformation of the cell wall, prompting cell wall‐remodeling enzymes that enable or disable existing cross‐links between layers.^[^ ^117^, ^118^, ^119^ ^]^ It was suggested that hyperosmotic shock triggers the compression of β‐glucan coils and the slippage of β‐glucan and chitin layers, leading to reduced cell wall elasticity and enhanced osmotic stress resistance.^[^ ^120^ ^]^ Likewise, cell wall elasticity remodeling was observed after caspofungin‐induced β‐glucan synthesis inhibition and GPI‐modified protein gene deletion.^[^ ^121^ ^]^

Many studies, both in mycology and biotechnology, are aimed at optimal production, usually involving high yields, for example, of biomass extracts, or minimal material loss, for instance, of cellulose, as opposed to lignin. Along these lines, Pelkmans et al. employed genetic engineering to alter fungal transcription factors in *S. commune*, promoting vegetative growth and yielding up to 2.8‐fold more biomass compared to the wild‐type strain.^[^ ^122^ ^]^ Furthermore, Yoav et al. achieved tunable lignin/cellulose degradation ratios in *P. ostreatus* through secretome composition genetic modification.^[^ ^123^ ^]^ This result is highly relevant in materials applications, since lower cellulose losses can increase mechanical performance of the overall biocomposite and unlock potential applications, such as in a biological pretreatment of biomass for bioethanol production.^[^ ^123^ ^]^

Again, it is important to note that the relationships between material outcomes and induced changes in fungal metabolism aren't necessarily straightforward considering all that remains unknown about the chemistry and interactions of cell wall polysaccharides and proteins and how they are interconnected. For example, although chitin is a structural material in the cell wall, the increase in chitin content may not necessarily lead to an increase in mycelial strength, as this may be accompanied by other changes such as the deregulation of β‐(1,3) glucan synthesis.^[^ ^113^ ^]^ Indeed, it is difficult to imagine how the rerouting of resources toward one particular outcome, like increased chitin in the cell wall, will not involve a trade‐off or loss elsewhere.

## 6 Balance of Nutrition and Non‐Nutrition

As previously mentioned, the fungal cell wall is not inert, rather, the cell wall responds to its environment of growth and alters both its composition and structure accordingly.^[^ ^124^, ^125^ ^]^ Fungal growth conditions can affect the localization of cellular components. For example, α(1‐3)‐glucans are typically found in the middle layer of fungal cells under agitated and submerged conditions, but they tend to be located in the outer layer under aerial conditions.^[^ ^126^, ^127^ ^]^ Similarly, diet can be used to modify properties, aka a “you are what you eat” approach, and the inclusion of non‐nutritive or recalcitrant elements can serve as stressors, inducing changes similar both in scale and in function to those discussed previously in the section on genetics and cell wall composition. Non‐nutritive but oftentimes functional elements can be bundled together within a lignocellulose substrate, e.g., silica in rice hulls,^[^ ^128^ ^]^ or included as separate components to the main source of nutrition, e.g., glass fines,^[^ ^129^ ^]^ montmorillonite nanoclays,^[^ ^130^ ^]^ or nanocellulose,^[^ ^88^ ^]^ with the goal of tailoring the performance of end materials.

It seems that as long as the basic conditions needed for growth are satisfied, the inclusion of non‐nutritive elements to achieve a target functionality is a good materials design strategy, as long as the levels are non‐toxic to the fungi. Considering biomass yield for chitin production, Jones et al. compared the submerged fermentation of *T. versicolor* using agricultural residues that were either finely ground solids (rice hulls, rich in silica) or solubilized (blackstrap molasses), without additional carbon supplementation, concluding that the solid substrate alone was insufficient for the growth of mycelial biomass.^[^ ^128^ ^]^ This work considered the quantity of mycelium, not its quality or chitin content, and the results may reflect differences in substrate availability or accessibility and lag times in growth with more difficult to digest substrates. Elsackar et al. concluded that myco‐composite mechanical performance depended more on the form of lignocellulose fiber substrates than on their chemical composition,^[^ ^131^ ^]^ suggesting that the balance of nutrition to non‐nutrition isn't critical, though a minimum threshold of nutrition and accessibility to that nutrition are certainly required for viability and fecundity. Jones et al. introduced high silicate substrates in the form of rice hulls (15‐20% silica) or glass fines (70‐90% silica) combined with wheat grains to achieve flame retardant composites.^[^ ^129^ ^]^ The inclusion of montmorillonite (MTM) nanoclays in a *T. versicolor*‐hemp pairing resulted in stunted growth, with the resulting composites exhibiting poor mechanical properties,^[^ ^130^ ^]^ further emphasizing the limitations and trade‐offs that can occur when non‐nutritive (albeit potentially functional) elements are included within the growth medium.

Indeed, one of the many impressive characteristics of fungi is their ability to adapt to the substrate that they are exposed to, even if it includes non‐nutritious or even toxic compounds, such as organic molecules, heavy metals, or metalloids. While some metals, such as sodium, potassium, calcium, magnesium, manganese, nickel, chromium, cobalt, copper, iron, and zinc, play an integral role in the life processes of fungi, others like silver, cadmium, lead, and mercury are non‐essential and potentially toxic.^[^ ^132^ ^]^ The toxicity of non‐essential metals occurs by the displacement of essential metals from their native binding sites or through interactions with essential biomolecules.^[^ ^67^ ^]^ However, at sufficiently high concentrations, both essential and non‐essential metals can damage the microbial cell membrane, disrupt cellular activities, alter enzyme function, and damage the structure of DNA.^[^ ^133^ ^]^ Therefore, fungi have developed myco‐remediation strategies as a defense mechanism against metal toxicity,^[^ ^68^ ^]^ which, from a materials standpoint, can also be used as a way to include metals into fungal materials, for instance to create conductive polymers, heat‐resistant materials, antimicrobial materials, ion exchange membranes, or other membranes, etc., Still, when fungal resources are rediverted to neutralize external threats like toxic metals, it seems likely that some other developmental aspect may be compromised, and at the same time, rather unlikely that the final state that is developed from a complex, interconnected living system could ever be approximated as a sum of individual parts.

Furthermore, by utilizing these adaptation pathways, fungi can synthesize metal nanoparticles through a growth‐driven assembly that mitigates the toxicity of metal ions by reducing them to pure metals. Simply put, they make use of electrostatics and adhesive proteins to trap and stabilize metal ions in the cell wall, reduce these ions by enzymes, and promote the nucleation and growth of metal intra‐ or extracellular nanoparticles stabilized by proteins.^[^ ^134^ ^]^ Microbially synthesized extracellular nanoparticles have the advantage of easy recovery, whereas intracellularly synthesized nanoparticles are reported to have lower polydispersity.^[^ ^135^ ^]^ This natural strategy to mitigate the toxicity of metal ions can be exploited in materials applications, for instance related to nanoparticle synthesis and shape control. Along this vein, gold nanoparticles were assembled into conductive microwires using *A. niger* hyphae as a template.^[^ ^136^ ^]^ In this case, glutamate was used as a nitrogen source and for the reduction of chloroauric acid to gold.^[^ ^136^ ^]^

In different environments, fungi are also known to induce the precipitation of a wide range of minerals in a process called biomineralization. Various species of fungi have been shown to facilitate calcium carbonate mineralization, such as was demonstrated by Zhang et al., who used the *Fusarium oxysporum* (*F. oxysporum*) mycelium network as a nucleation site for CaCO_3_ precipitation, resulting in self‐healing mycelium‐bound biocomposites with high water tightness.^[^ ^137^ ^]^ Similarly, Livne et al., used inactivated *T. versicolor* mycelium to synthetically induce CaCO_3_ needle (aragonite) precipitation.^[^ ^138^ ^]^ Different from the previous examples discussed in this section, aragonite biomineralization is unrelated to diet and development, since the fungal growth was terminated before the in vitro crystallization experiment.^[^ ^138^ ^]^ However, we include this example here as it relates to the interactions of fungi and non‐nutritious chemicals, with implications for possible novel organic‐inorganic materials applications.

In addition to including non‐nutrition, feeding fungi with harder‐to‐digest nutrition can be a tactic to tailor cell wall composition and properties.^[^ ^87^, ^88^ ^]^ Indeed, the presence of more easily digestible nutrients, such as glucose, has been shown to diminish lignocellulolytic enzyme secretion, thereby altering the interactions of the fungi with the substrate and yielding different overall properties.^[^ ^139^ ^]^ *T. versicolor* grown in a substrate composed of hemp fibers supplemented with bacterial cellulose (BC) was promising in regards to mechanical property tuning, although the solid content of BC used in this work was not specified and it is unclear whether the hemp or BC was preferentially digested or whether the enhancement is related to intact BC within the composite, an increase in mycelial mass, or something else.^[^ ^140^ ^]^

Nutritional conditions can also alter observed hyphal types, although this feature was not explicitly addressed in the BC example mentioned above. Attias and Abitbol et al. studied the submerged fermentation of *Trametes ochracea* (*T. ochracea)*, with highly crystalline, recalcitrant nanocellulose (NC) included in the growth medium, side by side with plentiful, readily digestible nutrition in the form of glucose.^[^ ^88^ ^]^ Mycelium development and hyphal morphologies were affected by the type and concentration of the NC included within the growth medium.^[^ ^88^ ^]^ With carboxymethylated cellulose nanofibrils (CNF), comparing 0.2 and 2 wt% concentrations of NC in the media, mycelium development occurred as discrete pellets and as a pellicle, respectively, and individual hyphae as nearly native‐like in appearance for the lower concentration, in contrast to spaghetti‐like, smooth, uniform, and long at the higher concentration, with these changes perhaps mostly related to the different viscosity and mass transport properties of the media.^[^ ^88^ ^]^ Compared with the hyphae from pure mycelium, closer examination of the materials resulting from the fermentation that included 0.2 wt% CNF indicated hyphae that were more fibrillar, regular, stiffer, and flatter, as well as an apparently diminished secretion of exopolysaccharides (EPS), possibly related to a densified cell wall. It was suggested that CNF may have become integrated within the fungal cell wall, or that some other alteration of the cell wall led to the observed effects, where the introduction of stiff, axisymmetric nanoparticles translated into stiffer biomaterials, intriguingly also obtained at higher biomass yield.^[^ ^88^ ^]^

In an earlier study by Haneef et al., hyphal morphology was also found to be affected by diet, with more collapsed hyphae observed when fed a harder‐to‐digest substrate consisting of microcrystalline cellulose (MCC) compared to a diet that was additionally supplemented with readily digestible nutrition (PDB).^[^ ^87^ ^]^ In this same study, the resulting pure fungal mycelium films were found to be enriched in chitin, corresponding to a higher Young's modulus and lower elongation at break.^[^ ^87^ ^]^ Similarly, Bulik et al. reported a 3‐4‐fold increase in the cell wall chitin levels of *S. cerevisae* as a result of glucosamine supplementation.^[^ ^115^ ^]^ As for the glucan content, Yousefi et al. posited that the naturally‐occurring covalent bonds between glucan and chitin act as a flexible matrix that improves mechanical and film formation properties.^[^ ^101^ ^]^ Since β−1,3 glucan synthase was suppressed when limiting the glucose concentration of *A. fumigatus*,^[^ ^141^ ^]^ strategies for improving material ductility could involve consideration of easily digestible nutrients. Haneef et al. also concluded that higher lipid and protein contents in the cell wall was related to easily digestible nutrition imparting a plasticizing effect, resulting in a more ductile myco‐material.^[^ ^87^ ^]^ While investigating the degradation of plastics by *P. chrysosporium*, Wu et al. observed increased secretion of hydrophobic proteins during PS and PLA colonization, the latter also producing more lipids.^[^ ^91^ ^]^ At the risk of oversimplification, there seem to be links between the morphological and chemical characteristics of the food and the properties of the resulting end materials, perhaps pointing out levers that can be turned to modify mycelium material properties toward, for example, strength, ductility, or hydrophobicity.

In the above examples, substrates rich in non‐nutritive components (e.g., MTM and rice hulls) interfere with mycelium development, whereas the replacement of easily digestible nutrients with recalcitrant ones (e.g., PDB replaced by MCC) or the addition of mechanically reinforcing and potentially digestible additives (e.g., BC) enhance certain properties. Of these studies mentioned, only in the NC‐*T. ochracea* work by Attias and Abitbol et al. was the media supplemented with glucose in order to preserve the crystalline nanoparticles within the end composite, or was the impact of growth environment on EPS production considered.^[^ ^88^ ^]^ Supplementation with easily digestible nutrients such as glucose may be important if the aim is to support the healthy growth of mycelium as well as to retain the functionality of the additive within the final composite, especially if the additive is lignocellulosic. Additionally, with the exception of the examples using NC, MTM, and gold salts,^[^ ^88^, ^130^ ^]^ the composites discussed above used non‐dispersible additives, which can compromise material uniformity especially if considering submerged fermentation.

## 7 Other Environmental Conditions

Aside from toxins and indigestible or recalcitrant nutrition, other aspects of the growth environment can stress fungi. For example, in multiple fungal species, chitin synthases become more active under cell wall stress conditions such as hypoxia,^[^ ^142^ ^]^ low osmotic strength,^[^ ^143^ ^]^ high temperature conditions,^[^ ^144^ ^]^ and changes in carbon source.^[^ ^115^ ^]^

Alteration of environmental stimuli can be useful to control process efficiency and yield since fungi are able to adapt their growth style and speed in response to external cues. For instance, using *Candida albicans (C. albicans*), Nadeem et al. demonstrated that changes in external pH and incubation temperature could be used to favor either yeast or hyphal (filamentous) forms, which affect growth and substrate adherence,^[^ ^50^ ^]^ and could therefore influence material uniformity. Pelletier et al. demonstrated that higher incubation temperatures between 30—35 °C enabled accelerated growth and inhibited the development of fruiting bodies, resulting in stronger and more homogeneous pure mycelium cell foam boards, as well as a more efficient and less contamination‐prone incubation stage.^[^ ^145^ ^]^ Numerous studies have also shown how fungi can adapt to extreme conditions,^[^ ^39^, ^46^, ^49^, ^61^, ^62^ ^]^ since they have the ability to optimize nutrient acquisition and adjust their growth rate depending on nutrient and resource availability, such as light,^[^ ^47^ ^]^ gravity,^[^ ^61^ ^]^ and chemical gradients,^[^ ^58^ ^]^ all of which could be exploited to tune fungal growth for myco‐materials. One example is the work by Pelletier et al., where CO_2_ and O_2_ gradients in the incubation chamber were used to guide fungal propagation into void spaces, thus yielding pure mycelium foams that were homogeneous and free from growth media.^[^ ^145^ ^]^ Fungi can also alter their metabolic pathways to better adapt to changing environmental conditions, which allows them to adjust their energy production and nutrient utilization to better suit their current conditions. For instance, many transcribed genes in fungi were found to exhibit a light response within a matter of minutes, and this response is affected by the carbon source upon which the fungi is cultivated.^[^ ^48^ ^]^

In response to environmental cues, fungi can also produce a diverse range of secondary metabolites that enable them to interact with their environment, such as antimicrobial and antifungal compounds that help them compete for resources.^[^ ^66^ ^]^ Although already exploited in the pharmaceutical industry,^[^ ^146^ ^]^ this feature could be highly beneficial in materials‐oriented applications where antimicrobial functions are needed, such as biomedical devices or antibacterial applications.

## 8 Conclusion and Future Perspectives

While there is already an extensive research body on myco‐materials, many knowledge gaps have been identified by ourselves and others.^[^ ^10^, ^78^, ^147^, ^148^ ^]^ The focus of this perspective is how fungal genetics, including genetic modifications and conditions of growth can lead to fundamental changes, such as changes in cell wall composition, hyphal morphology, metabolic pathways, etc., which in turn can influence the properties of materials that are derived from fungal growth. We highlight below a few of these gaps that relate directly to the focus of this perspective.

A lot is known about fungal metabolism and adaptation, and while many principles may be universal, few studies have specifically addressed the species most often used in mycelium materials production, usually wood‐rotting fungi, to confirm their characteristics. Therefore, future research might be directed in two ways: first, by conducting detailed studies on common species used in mycelium materials to confirm their adaptation. Second, by expanding the research area to other fungal species that have been less studied, are non‐toxic, and have potential utility in the development of fungal‐based materials. This research will ideally explore the different aspects discussed here, specifically fungal species, genetics, balance of nutrition and non‐nutrition, and growth environment. It is likely that conditions of nutrition and growth impact fungal development in a species‐dependent manner, where some fungal species may be more resilient to extreme conditions than others. We recognize however that it is non‐trivial to fill in all the gaps on how a given species (with its specific genetic code) adapts to the vast possibilities of nutrition and environmental cues. Indeed, it seems beyond the scope of materials science, and perhaps instead of taking on the daunting challenge of understanding all aspects of nature versus nurture, materials scientists in this field might more reasonably be tasked with selecting nutrition and growth environments that support different material aims, asking ourselves: What is our aim? Are we designing a new food? A flame‐retardant foam? An insulation material? What substrate makes sense and is available from a resource vantage? Can we redirect waste into a new product? What are the best ways to process the material? By considering these practicalities, it can be possible to create new and improved functional materials, perhaps later asking how substrate or other aspects of environment leveraged the adaptive behaviors of fungi and manifested in the resulting materials? By answering these types of questions as we develop new myco‐materials, we can begin to make strong links between mycelium properties and the many possible aspects that may influence these properties, eventually leading to improved materials design strategies.

It is also a challenge within the field of materials development that optimal growth conditions, considering features such as biomass yield, composition, quality, and growth times, are usually not known, and neither are the minimum conditions required for viability. This makes sense considering the vastness of the process variable space; however, it does add a layer of complication for the use of fungi as a source of materials, in that most studies do not optimize the set of growth conditions that are used toward achieving the desired outcome in any meaningful way, and although it is the approach taken by many researchers, comparisons of different species using the same set of conditions are not necessarily justified. Indeed, the response of fungi to changes, especially if they are combined, is not likely to be the simple sum of individual stress responses. In complex environments, such as those described in this perspective, the response and adaptation of fungi are not easily predicted.^[^ ^149^ ^]^ Therefore, more sophisticated methods are warranted in order to understand the complex interactions of factors, including genetics, that affect the final properties of engineered materials. In this regard, useful strategies could include computational methods both to design the experiments (such as Design of Experiments techniques or artificial intelligence models) and to analyze correlations.

Moreover, even for species whose growth mechanisms are already extensively documented, we may still lack understanding of how mycelium composition and structure relate to material properties, which is fundamental to contextualize fungal biomaterials within the fields of materials science and engineering. Generally, the work that has been performed in this area considers the quantity of mycelium, not its quality or composition, and perhaps a more in‐depth understanding of the different outcomes could improve the understanding of the tunability potential of myco‐materials when exposed to nutrient‐ and environment‐induced stressors. Additionally, since fungi are living organisms, the matter of reproducibility is also elusive, since the biological factors involved in fungal adaptation can add a layer of complexity in terms of obtaining controlled outcomes. Many studies do not indicate or control for how far removed the mycelium inoculum is from the mother culture, with genetic variability due to mutation potentially showing up in later generations. Is there a generational distance from the mother culture where the cultivated species deviates to an extent that matters? Perhaps this is something to address in the subsequent literature.

Finally, scaling up of the process is vital for the commercial availability of these myco‐materials, and standardized production processes must be defined in order to facilitate knowledge transfer. Fungal growth for materials as outlined in Figure 2, while generally having low energy and other resource demands, requires time and physical space. We have rarely seen reports that address feasibility, indicating for example, what percent of the market share for a given application area could reasonably be replaced with a fungal material alternative? Considering the scale, some applications may emerge as more relevant than others. As the field continues to develop, it will be exciting to witness the emergence of the fields of impact, whether as an alternative protein for food or leather or insulating foam.

## Conflict of Interest

The authors declare no conflict of interest.
